# Hydrolysis and Enantiodiscrimination of (*R*)- and (*S*)-Oxazepam Hemisuccinate by Methylated β-Cyclodextrins: An NMR Investigation

**DOI:** 10.3390/molecules26216347

**Published:** 2021-10-20

**Authors:** Andrea Cesari, Federica Balzano, Gloria Uccello Barretta, Alessandra Recchimurzo

**Affiliations:** 1 Dipartimento di Scienze Chimiche, Università di Padova, Via Marzolo 1, 35131 Padova, Italy; andrea.cesari@unipd.it; 2 Dipartimento di Chimica e Chimica Industriale, Università di Pisa, Via Moruzzi 13, 56124 Pisa, Italy; alessandra.recchimurzo@phd.unipi.it

**Keywords:** NMR, chiral discrimination, methylated cyclodextrins, benzodiazepines, hydrolysis, inclusion complexes

## Abstract

Partially and exhaustively methylated β-cyclodextrins [(2-methyl)-β-CD (MCD), heptakis-(2,6-di-*O*-methyl)-β-CD (DIMEB), and heptakis-(2,3,6-tri-*O*-methyl)-β-CD (TRIMEB)] have been compared in the hydrolysis and enantiodiscrimination of benzodiazepine derivative (*R*)- or (*S*)-oxazepam hemisuccinate (OXEMIS), using nuclear magnetic resonance (NMR) spectroscopy as an investigation tool. After 6 h, MCD induced an 11% hydrolysis of OXEMIS, remarkably lower in comparison with underivatized β-CD (48%), whereas no hydrolysis was detected in the presence of DIMEB or TRIMEB after 24 h. DIMEB showed greater ability to differentiate OXEMIS enantiomers in comparison to TRIMEB, by contrast MCD did not produce any splitting of racemic OXEMIS resonances. Both enantiomers of OXEMIS underwent deep inclusion of their phenyl pendant into cyclodextrins cavities from their wider rims, but tighter complexes were formed by DIMEB with respect to TRIMEB.

## 1. Introduction

Benzodiazepines (BDZs) are a class of psychopharmaceuticals employed as sedatives, anxiolytics, hypnotic, and anticonvulsants with less collateral effects than barbiturate. The main biological mechanism of BDZs involves a positive allosteric modulation of GABA_A_ receptors located in the brain, enhancing the effect of γ-aminobutyric acid neurotransmitter, and hence reducing the excitability of neurons [1].

**Figure 1:**
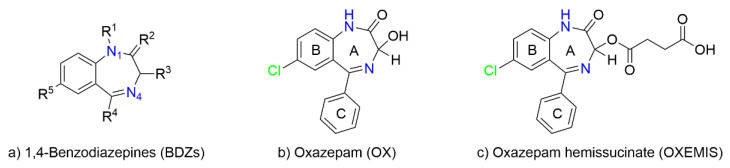
Chemical structures of (**a**) Diazepam (*Valium*): R^1^=CH_3_, R^2^=O, R^3^=H, R^4^=phenyl, R^5^=Cl; Alprazolam (*Xanax*): R^1^=CH(CH_3_)=N-N=R^2^, R^3^=H, R^4^=phenyl, R^5^=Cl; Flurazepam (*Dolmane*): R^1^=(CH_2_)_2_N(Et)_2_, R^2^=O, R^3^=H, R^4^=2-fluorophenyl, R^5^=Cl; Lorazepam (*Ativan*): R^1^=H, R^2^=O, R^3^=OH, R^4^=2-chlorophenyl, R^5^=Cl; Clonazepam (*Klonopin*): R^1^=H, R^2^=O, R^3^=H, R^4^=2-chlorophenyl, R^5^=NO_2_. (**b**) Oxazepam (*Serax*), and (**c**) Oxazepam succinate half-ester (hemisuccinate).

The skeletal core of BDZs is composed of a benzene ring fused with a 1,4-diazepine (or a 1,5-diazepine) and decorated with different substituents and/or additional heteroaromatic rings (examples of BDZs are given in Figure 1a). Despite BDZs have the same biological target, the variety of their chemical structures reflects the diverse pharmacokinetics (short-, intermediate-, and long-acting drugs), which can be addressed for the treatment of different medical conditions [2].

Among commonly used BDZs, Oxazepam (OX, Figure 1b), which has been prescribed for curing insomnia and anxiety arising from alcohol withdrawal syndrome, is a chiral BDZ with an asymmetric center at C_3_-OH, but it is commercialized as a racemic mixture. However, some studies have reported a different activity of (*S*)-OX with respect to (*R*)-OX, with the former from 100- to 200-fold more affine to the binding site in comparison with the latter one [3]. Therefore, administering low amounts of the more active stereoisomer can be the way to reduce unwanted side effects. Moreover, there are evidence that hepatic glucuronidation (main route of human clearance) produces enriched (*S*)/(*R*)-OX-glucuronide diastereomeric ratios, as found in human plasma and urinary excretions [4,5]. This metabolic difference is not common to all BDZs [6]. The stereoselectivity of glucuronosyltransferase isoenzymes can be also interfered by other administered drugs [7].

Attempts of separation of BDZs enantiomers are made difficult by the occurrence of racemization equilibria in enantioseparation conditions (aqueous medium). The inversion of the chiral center occurs very rapidly in vitro but the situation might be different in vivo, since the strong stereospecific binding to human serum albumin can be involved [8]. Recently, four different mechanisms of racemization of Oxazepam have been proposed and compared with a DFT approach, denoting the ring-chain tautomerization as the best fitting mechanism of the experimental data available [9].

To avoid rapid racemization of BDZs during separation processes, the synthesis of their stereochemically stable and more soluble ester derivatives has been proposed [10,11]. Interestingly, conjugation of oxazepam hemisuccinate (OXEMIS, Figure 1c) with dopamine improved the neurotransmitter delivery into the brain and enhanced GABAergic transmission [12]. Enantioseparation of BDZs hemisuccinates was achieved by using cyclodextrins (CDs)-based chromatographic column for HPLC [13]. As a matter of fact, interaction at the apolar cavity of cyclodextrins constitutes a privileged interaction mechanism with several kinds of substrates leading to the inclusion of their apolar moieties [14,15,16,17,18]. Moreover, being cyclodextrins chiral, the formation of diastereomeric inclusion complexes produces potentially differentiable signals for the two included enantiomers by chromatographic [19,20,21,22,23,24,25] and/or spectroscopic techniques [24,25,26,27,28]. Commonly, inclusion does not involve polar pendants of enantiomeric substrates which may protrude from the large and small rims of the cyclodextrins and interact with cyclodextrin hydroxyl functions incurring into hydrolytic processes, which have been reported for ester compounds [29,30,31].

In consideration of above premises, we focused our attention on partially and exhaustively methylated β-cyclodextrins [(2-methyl)-β-CD (MCD), heptakis-(2,6-di-*O*-methyl)-β-CD (DIMEB), and heptakis-(2,3,6-tri-*O*-methyl)-β-CD (TRIMEB)] to be compared to native β-CD with regard to the ability to differentiate OXEMIS enantiomers and propensity to hydrolyze their ester function. Both aspects were investigated by nuclear magnetic resonance (NMR) spectroscopy. As a matter of fact, CDs are very popular chiral solvating agents (CSAs) for NMR, which have been employed to differentiate NMR signals of several classes of enantiomeric substrates [26,27,28]. Furthermore, quantitative response of NMR makes this technique very appealing both for the determination of enantiomeric excesses and for the detection and quantification of degradation products. Finally, enormous potentialities of NMR in the field of investigations of complexation phenomena have been exploited to investigate the interaction mechanisms involving the cyclodextrin-based chiral auxiliaries and enantiomeric OXEMIS at a molecular level and, hence, ultimately, the chiral discrimination processes.

## 2. Results and Discussion

### 2.1. OXEMIS Characterization

**Figure 2:**
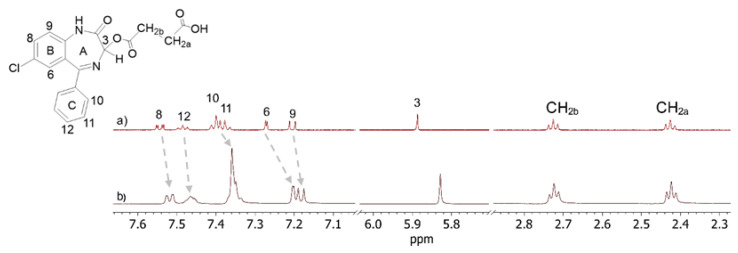
^1^H NMR (600 MHz, K_2_HPO_4_/D_2_O 50 mM, 25 °C) spectra of OXEMIS: (**a**) 1 mM and (**b**) 12 mM.

^1^H NMR spectrum of OXEMIS (1 mM, Figure 2a) showed three sets of signals. The aromatic protons resonances between 7.1 and 7.6 ppm were easily distinguished on the basis of multiplicity, while H_3_ was the only isolated singlet clearly observable at 5.89 ppm. Methylene group adjacent to the chiral center at C_3_ (CH_2b_, 2.73 ppm) was differentiated from CH_2a_ (2.43 ppm) on the basis of the ROE effect produced at the frequency of H_3_ (Figure S1a, Supplementary Material). The dependence of ^1^H NMR chemical shifts of OXEMIS on concentration witnessed the presence of self-association processes (Figure 2a,b), which could in principle play a relevant role in the hetero-complexation behavior.

**Figure 3:**
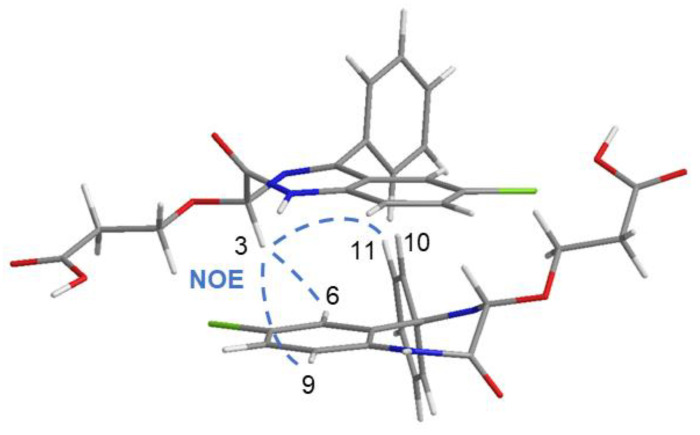
3D representation of OXEMIS dimer as obtained from NMR data.

Low solubility of OXEMIS allowed to investigate only the 13.7 mM to 0.3 mM concentration gradient, inside which the greatest chemical shift variations (Δδ = δ_[13.7 mM]_ − δ_[0.3 mM]_) were detected for H_3_/H_9_ (≈−27 Hz) and H_6_/H_8_ (≈−12 Hz) (Figure S2, Supplementary Material). Protons of the lateral chain (CH_2a_/CH_2b_) were affected to a less extent. Interestingly, every proton underwent low-frequency shifts with concentration increasing, hinting a π-π stacking-like self-association of OXEMIS, which was witnessed by the nature of dipolar correlations detected in the 2D ROESY map (Figure S1b, Supplementary Material). As a matter of fact, H_3_ produced unexpected ROEs at the frequencies of H_10_/H_11_ (C-ring) and H_6_/H_9_ (B-ring). A representation of self-associated OXEMIS is shown in Figure 3.

An attempt to calculate self-association constant (K_a_), based on the non-linear fitting of dilution data, failed due to the fact that limited solubility of OXEMIS did not allow to explore a sufficiently wide range of concentrations. Indeed, an almost linear dependence of δ_obs_ on concentration was found (Figure S3a, Supplementary Material), leading to great errors in K_a_ determination. On the other hand, a quite small K_a_ was calculated in CDCl_3_ (K_a_ = 5.1 ± 0.2 M^−1^) where a wider range of concentrations could be explored (0.5 to 132 mM), as shown in Figure S3b, Supplementary Material. It is noteworthy that, based on such K value, the molar fraction of dimer was less than 3%, and hence negligible, in the concentration conditions (6 mM) we employed for the investigation of OXEMIS complexation by cyclodextrins.

### 2.2. CDs Characterization

**Figure 4:**
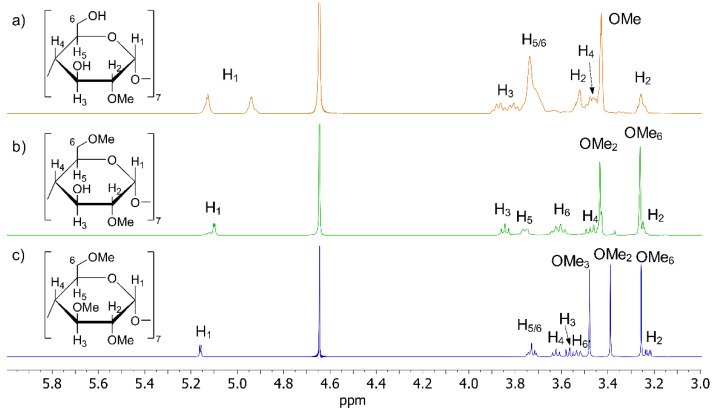
^1^H NMR (600 MHz, K_2_HPO_4_/D_2_O 50 mM, 25 °C, 12 mM) spectra of: (**a**) MCD, (**b**) DIMEB, and (**c**) TRIMEB.

The three cyclodextrins candidates for OXEMIS enantiodiscrimination were MCD, DIMEB, and TRIMEB (^1^H NMR spectra shown in Figure 4a–c).

MCD is a cyclodextrin derivatized at OH_2_, with a substitution degree of 3.5, as carefully determined in the ^1^H NMR spectrum by comparing the integrated areas of the anomeric protons resonances between 5.3 to 4.9 ppm and the superimposed proton signals of ring and methoxy groups in the spectral region 4.0 to 3.2 ppm. Two kinds of anomeric protons were distinguished, which originated by non-methylated (4.94 ppm) and methylated (5.13 ppm) glucopyranose units. As a matter of fact, selective perturbation at the frequency of 5.13 ppm resulted in the expected ROE effect at the frequency of the methoxy group (see [32] for a detailed characterization of MCD).

In the ^1^H NMR spectrum of DIMEB (Figure 4b), two singlets attributable to methoxy groups were clearly identified at 3.44 ppm and 3.27 ppm. Comparison of chemical shifts of DIMEB and native β-CD (Table 1) showed that major differences between their chemical shifts were found for H_1_, H_2_, and H_6_ protons, as expected for a 2,6-disubstitution. Perturbation at the frequency of anomeric proton H_1_ at 5.11 ppm produced ROE at 3.44 ppm, which was attributed to OMe_2_ (Figure S4, Supplementary Material), thus allowing to attribute OMe_6_ at 3.27 ppm. The ratio between integrated areas of H_1_ and ring protons (including methoxy signals) confirmed the presence of two methoxy groups per glucopyranose unit of DIMEB (DS ≈ 14). Remaining ring protons were assigned (Table 1) by 1D ROESY, 2D COSY, and 2D HSQC experiments (Figures S5–S7, Supplementary Material).

TRIMEB produced a single set of well-resolved signals (Figure 4c), resonances of which were attributed (Table 1) by comparing scalar and dipolar interactions detected by 1D ROESY, 2D COSY, and 2D HSQC experiments (Figures S8–S10, Supplementary Material).

### 2.3. OXEMIS Hydrolysis by Cyclodextrins

**Figure 5:**
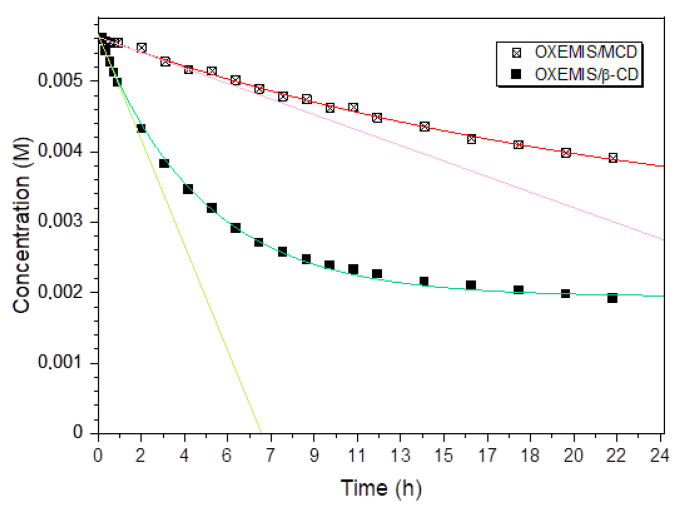
OXEMIS concentration in the presence of 2-equivalents of β-CD or MCD (12 mM) in D_2_O (K_2_HPO_4_ 50 mM) over time, as detected by ^1^H NMR (600 MHz, 25 °C).

The chemical stability of OXEMIS over time was compared as pure compound and in the presence of 2 equivalents of each cyclodextrin, in buffered solutions (K_2_HPO_4_/D_2_O, 50 mM). OXEMIS hydrolyzation was followed directly in the NMR tube (T = 25 °C) and in static conditions. The resonance of CH_2a_ group located on hemisuccinic chain was selected for monitoring the process. The parent cyclodextrin β-CD was included for comparison as well. Less than 1% of hydrolysis product (OX) was detected after 6 h in the case of pure OXEMIS, whereas a rapid hydrolyzation was detected in the presence of β-CD, with an exponential decay during the very first points of the kinetic (first 2 h, Figure 5). Hydrolysis was observed even in the presence of MCD, but with a remarkably slower rate (Figure 5). As an example, after 6 h, the concentration of hydrolysis product in presence of β-CD was five-fold (48%) than that detected in the presence of MCD (11%).

Therefore, the hydrolysis of OXEMIS is correlated with the availability of free reactive -OH groups in the cyclodextrin, able to establish hydrogen bonds, probably with the hemisuccinic pendant. Accordingly, DIMEB and TRIMEB did not produce hydrolysis within the 24 h, pointing out the relevance of the interaction of hemisuccinic chain with the hydroxyls at the C_2_ and C_6_ sites.

### 2.4. OXEMIS Enantiodiscrimination

**Figure 6:**
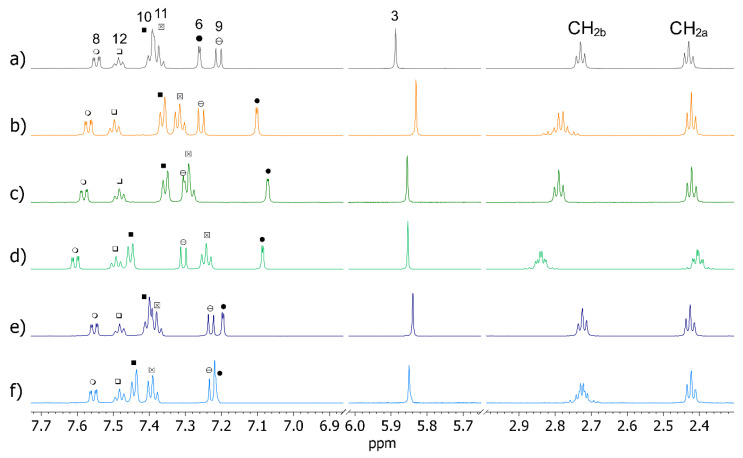
^1^H NMR (600 MHz, K_2_HPO_4_/D_2_O 50 mM, 25 °C) spectra of: (**a**) pure OXEMIS (6 mM), (**b**) OXEMIS/MCD (1:2), (**c**) (*R*)-OXEMIS/DIMEB (1:2), (**d**) (*S*)-OXEMIS/DIMEB (1:2), (**e**) (*R*)-OXEMIS/TRIMEB (1:2), and (**f**) (*S*)-OXEMIS/TRIMEB (1:2).

When MCD was added to pure *rac*-OXEMIS, only variations in chemical shifts were detected without signal splitting (Figure 6a,b, Table 2). Hence, a complexation occurred which did not produce chiral discrimination.

By contrast, DIMEB produced complexation shifts (Δδ = δ_mixture_ − δ_pure_) along with enantiodifferentiation of several OXEMIS protons (Figure 6a,c,d). In particular, significant complexation shifts were measured for H_6_, H_9_, H_10_, and H_11_ (0.04 to 0.19 ppm, Table 2). Major non-equivalences (ΔΔδ = |Δδ_(*S*)_ − Δδ_(*R*)_|) were detected (Table 2) for the protons belonging to ring C and for the methylene group CH_2b_ near to the ester moiety. Interestingly, (*S*)-enantiomer protons were more affected by the presence of DIMEB, being its complexation shifts greater than those of (*R*)-enantiomer, with the sole exception of proton H_6_. It is noteworthy that, in each case, internal protons of DIMEB were more affected by the presence of OXEMIS than external ones were (Table 3), as a clear indication of the occurrence of inclusion phenomena.

In presence of TRIMEB, smaller complexation shifts and non-equivalences were detected (Table 2). In particular, methylene protons of the hemisuccinate fragment were almost unaffected by TRIMEB (Δδ = 0.00 to 0.01 ppm, Table 2). Correspondingly, small complexation shifts were also measured for TRIMEB protons (Table 3, Figure S11 in Supplementary Material), with the internal ones more affected, in particular in the mixture containing the (*S*)-enantiomer.

### 2.5. OXEMIS/CD Interaction Mechanism

To give a better understanding of the origin of chiral discrimination process of OXEMIS due to its interaction with DIMEB and TRIMEB, stoichiometries and association constants of the diastereomeric complexes were determined in mixtures containing each cyclodextrin and OXEMIS enantiomer. By using the Job method [33], both OXEMIS/DIMEB and OXEMIS/TRIMEB complexes showed 1-to-1 interaction stoichiometries for both the guest enantiomers (Figure S12, Supplementary Material). Association constants (K_a_) of the diastereomeric complexes were calculated by using the Foster-Fyfe method [34] in solutions containing a fixed amount of OXEMIS and increasing amounts of the cyclodextrin (Figure S13, Supplementary Material). (*S*)-OXEMIS formed a tighter complex with DIMEB with respect to (*R*)-OXEMIS (K_a_ = 930 ± 38 M^−1^ vs. 137 ± 11 M^−1^, respectively), whereas association constants for TRIMEB were significantly lower for both enantiomers (K_a_ = 40.5 ± 3.0 M^−1^ for (S)-OXEMIS vs. 2.5 ± 0.9 M^−1^ for (R)-OXEMIS).

**Figure 7:**
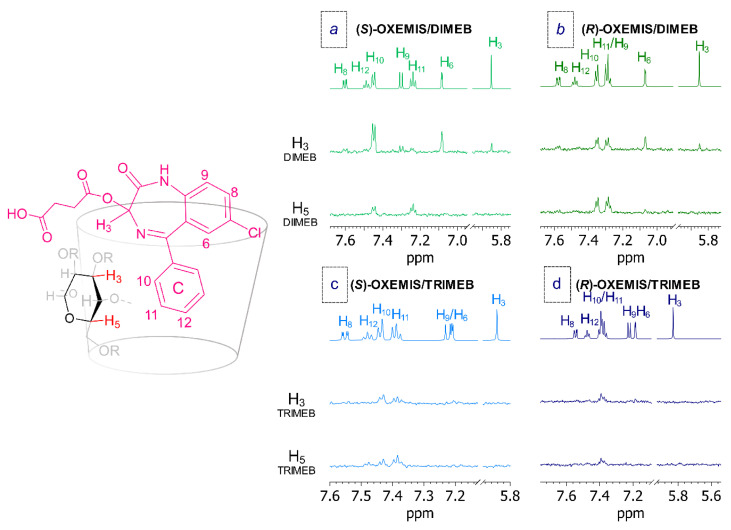
^1^H NMR (600 MHz, K_2_HPO_4_/D_2_O 50 mM, 25 °C) and 1D ROESY spectra (H_3_ and H_5_ protons) of cyclodextrins for: (**a**) (*S*)-OXEMIS/DIMEB (1:2), (**b**) (*R*)-OXEMIS/DIMEB (1:2), (**c**) (*S*)-OXEMIS/TRIMEB (1:2), and (**d**) (*R*)-OXEMIS/TRIMEB (1:2).

The interaction stereochemistry was investigated by 1D and 2D ROESY analyses. As shown in Figure 7a, intermolecular ROEs were detected between internal protons of DIMEB and phenyl C-ring protons along with H_6_ and H_3_ of (*S*)-OXEMIS. In particular, H_3_ protons of DIMEB (located on the internal wider rim) produced the most intense cross-peak on H_10_ and significant dipolar interactions with H_6_ and H_3_ protons of (*S*)-OXEMIS. By contrast, H_5_ protons of DIMEB (on the internal narrower rim) gave the main ROEs with H_11_ and H_10_ of phenyl C-ring. Such proximity constraints support a deep inclusion of phenyl C-ring of (*S*)-OXEMIS from the wider cavity of DIMEB, according to the most relevant complexation shifts measured for (*S*)-OXEMIS protons (phenyl C-ring, H_6_ and CH_2b_) and for internal protons (Δδ_H5_ > Δδ_H3_) of DIMEB (Table 2 and Table 3).

In the mixture (*R*)-OXEMIS/DIMEB analogous ROEs between internal protons of DIMEB and H_10_/H_11_ (phenyl C-ring), H_6_ and H_3_ of (*R*)-OXEMIS were detected (Figure 7b), but with lower intensities.

The analysis of (*S*)-OXEMIS/TRIMEB and (*R*)-OXEMIS/TRIMEB mixtures showed ROE patterns for phenyl C-ring similar to those observed in the presence of DIMEB (Figure 7), confirming the inclusion of ring C from the wider rim of TRIMEB. However, H_6_ and H_3_ protons of OXEMIS did not give ROE effects with internal protons of TRIMEB and H_12_ proton of phenyl C-ring was in spatial proximity of H_5_ protons of TRIMEB, in according to a less deep inclusion of the guest with respect to DIMEB complexes.

The relative magnitude of ROE effects for the two enantiomers in presence of DIMEB or TRIMEB well reflects the strength of OXEMIS/cyclodextrin interaction as quantified by association constants determination.

## 3. Materials and Methods

### 3.1. Materials

Heptakis-(2,6-di-*O*-methyl)-β-CD (DIMEB, 98%) and K_2_HPO_4_, β-Cyclodextrin (β-CD), heptakis-(2,3,6-tri-*O*-methyl)-β-CD (TRIMEB, 98%) were purchased from Sigma-Aldrich (St. Louis, MO, USA). Deuterated water (D_2_O) and deuterated chloroform (CDCl_3_) were purchased from Deutero GmbH (Kastellaun, Germany). Oxazepam (7-chloro-3-hydroxy-5-phenyl-1,3-dihydro-2H-1,4-benzodiazepin-2-one, OX) was kindly donated by Profarmaco Spa (Milan, Italy). Racemic hemisuccinate half-ester of Oxazepam was prepared by acylation with succinic anhydride in presence of pyridine according to [13]. The pure enantiomers of OX (enantiomeric excess (e.e.) >98%, determined by HPLC on β-cyclodextrin-based CSP—Cyclobond I) were obtained by fractional crystallization (acetone/water 100:1) of diastereomeric salts with (−)-(1*R*,2*S*)-ephedrine.

### 3.2. NMR Experiments

NMR measurements were performed on Varian INOVA600 spectrometer (Varian Inc., Palo Alto, CA, USA) operating at 600 MHz for ^1^H, equipped with Varian temperature control unit (25 ± 0.1 °C). 2D NMR spectra were obtained by using standard sequences. Spectral width used was the minimum required in both dimensions. 2D gCOSY (gradient correlated spectroscopy) spectra were recorded in the absolute mode acquiring 8 scans with a 1 s relaxation delay between acquisitions and 4K data points for each of 200 FIDs. 2D TOCSY (total correlation spectroscopy) spectra were recorded acquiring 8 scans with a 1 s relaxation delay, 200 increments, 4K data points, and a mixing time of 120 ms. 1D ROESY (rotating-frame Overhauser enhancement spectroscopy) spectra were recorded with a mixing time of 0.3 s, a delay of 1 s, and 1024 scans. 2D ROESY map was recorded with mixing time of 0.3 s and 32 scans. gHSQC (gradient Heteronuclear Single Quantum Coherence) spectra were obtained with 1.2 s relaxation delay and 16 scans for each of the 128 increments.

### 3.3. NMR Samples Preparation for Stoichiometries and Association Constants

The NMR samples for stoichiometries determination were prepared by mixing different volumes of stock solutions of each component (8 mM) to a prefixed volume directly into the NMR tube.

The NMR samples for the determination of association constants of the diastereomeric complexes formed by (*S*)- and (*R*)-OXEMIS with DIMEB or TRIMEB were prepared at constant concentration of OXEMIS (0.2 mM) and CD/OXEMIS ratio ranging from 1 to 200.

## 4. Conclusions

Among methylated cyclodextrins, DIMEB was the more suited both to interact with the two enantiomers of OXEMIS and differentiate them, without hydrolysis of the hemisuccinic arm. Phenyl ring C of OXEMIS well fits in the cavity sizes both of DIMEB and TRIMEB, but enhanced stabilization of the two enantiomers inside the cavity of DIMEB is correlated to the presence of residual underivatized OH functions, probably due to their hydrogen bond interactions with the hemisuccinate pendant protruding from the cyclodextrin cavity. Degradation processes are mainly due to the presence of hydroxyl groups at C_2_ and C_6_, as found in the case of MCD and native β-CD, with a particular relevance of C_2_-hydroxyls.

## Figures and Tables

**Table 1:** ^1^H NMR (600 MHz, 12 mM, K_2_HPO_4_/D_2_O 50 mM, 25 °C) chemical shifts (ppm) of β-CD, MCD, DIMEB, and TRIMEB protons.

	β-CD	MCD ^1^	DIMEB	TRIMEB
H_1_	4.94	4.94/5.13	5.11	5.16
H_2_	3.52	3.53/3.26	3.26	3.22
H_3_	3.84	3.81/3.88	3.85	3.56
H_4_	3.46	3.45/3.48	3.47	3.62
H_5_	3.74	3.73/3.70	3.77	3.74
H_6/6′_	3.75	3.74/3.74	3.63	3.53/3.72
OMe_2_	-	3.44	3.44	3.39
OMe_3_	-	-	3.27	3.48
OMe_6_	-	-	-	3.25

^1^ Chemical shifts relative to non-methylated and 2-methylated MCD glucopyranose units, both present in MCD.

**Table 2:** ^1^H NMR (600 MHz, K_2_HPO_4_/D_2_O 50 mM, 25 °C) chemical shifts (ppm) of pure OXEMIS (6 mM) and complexation shifts (Δδ = δ_mixture_ − δ_pure_, ppm) of OXEMIS in mixture with 2-equivalents of MCD, DIMEB, and TRIMEB.

			OXEMIS/MCD	OXEMIS/DIMEB		OXEMIS/TRIMEB	
Ring		δ	Δδ	Δδ_(*S*)_	Δδ_(*R*)_	Δδ_(*S*)_	Δδ_(*R*)_
A	H_3_	5.89	−0.06	−0.03	−0.03	−0.05	−0.04
B	H_6_	7.25	−0.16	−0.18	−0.19	−0.04	−0.06
B	H_8_	7.54	0.02	0.06	0.03	0.00	0.00
B	H_9_	7.20	0.05	0.10	0.09	0.01	0.02
C	H_10_	7.40	−0.03	0.06	−0.04	0.05	0.01
C	H_11_	7.37	−0.05	−0.13	−0.08	0.02	0.01
C	H_12_	7.49	0.01	0.01	−0.01	−0.01	−0.01
-	CH_2a_	2.43	−0.01	−0.03	−0.01	−0.01	0.00
-	CH_2b_	2.73	0.05	0.11	0.06	−0.01	−0.01

**Table 3:** ^1^H NMR (600 MHz, K_2_HPO_4_/D_2_O 50 mM, 25 °C) complexation shifts (Δδ = δ_mixture_ − δ_pure_, ppm) of cyclodextrin protons (12 mM) in the presence of OXEMIS (6 mM).

	DIMEB/OXEMIS		TRIMEB/OXEMIS	
Proton	Δδ_(*S*)_	Δδ_(*R*)_	Δδ_(*S*)_	Δδ_(*R*)_
H_1_	−0.02	−0.02	−0.01	−0.01
H_2_	−0.03	0.00	−0.01	−0.01
H_3_	−0.09	−0.07	−0.02	−0.01
H_4_	−0.03	−0.01	−0.01	−0.01
H_5_	−0.16	−0.09	−0.03	−0.01
H_6/6′_	−0.02	−0.06	0.00	0.00
OMe_2_	0.00	0.00	−0.01	−0.01
OMe_3_	−0.01	−0.01	−0.03	−0.03
OMe_6_	-	-	0.00	0.00

## Data Availability

Data are included in this published article.

## Supplementary Materials

The following are available online: Figure S1: 2D ROESY map expansion of OXEMIS; Figure S2: Dependence of OXEMIS ^1^H NMR chemical shifts variations on concentration; Figure S3: Fitting of dilution data of OXEMIS in D_2_O and CDCl_3_; Figure S4: 1D ROESY and ^1^H NMR spectra of DIMEB; Figure S5: 1D ROESY spectrum of DIMEB for H1; Figure S6: 2D COSY map of DIMEB; Figure S7: 2D HSQC map of DIMEB; Figure S8: 1D ROESY spectrum of TRIMEB for H1; Figure S9: 2D COSY map of TRIMEB; Figure S10: 2D HSQC map of TRIMEB; Figure S11: ^1^H NMR spectra of pure DIMEB, (*R*)-OXEMIS/DIMEB, (*S*)-OXEMIS/DIMEB, pure TRIMEB, (*R*)-OXEMIS/TRIMEB, and (*S*)-OXEMIS/TRIMEB; Figure S12: Job plot of (*S*)-OXEMIS in the presence of TRIMEB; Figure S13: Foster-Fyfe determination of association constant for (*R*)- and (*S*)-OXEMIS with DIMEB and TRIMEB.

## Sample Availability

Not available.
