# Supplementary material for: Hydrolysis and Enantiodiscrimination of (R)- and (S)-Oxazepam Hemisuccinate by Methylated β-Cyclodextrins: An NMR Investigation
Source: Molecules. 2021 Oct 20;26(21):6347. doi: 10.3390/molecules26216347 (PMC8587842; doi:10.3390/molecules26216347)
Supplement: Supplementary file 1 [file molecules-26-06347-s001.zip › molecules-1411119 - supplementary.pdf]

Hydrolysis and enantiodiscrimination of (*R*)- and (*S*)-oxazepam hemisuccinate by methylated β-cyclodextrins: an NMR investigation

Andrea Cesari[1], *Federica Balzano*[2,*], *Gloria Uccello Barretta*[2,*] *and Alessandra Recchimurzo*[2]

1 Dipartimento di Scienze Chimiche, Università di Padova, Via Marzolo 1, 35131 Padova, Italia.

2 Dipartimento di Chimica e Chimica Industriale, Università di Pisa, via Moruzzi 13, 56124 Pisa, Italia.

* Correspondence: federica.balzano@unipi.it; gloria.uccello.barretta@unipi.it.

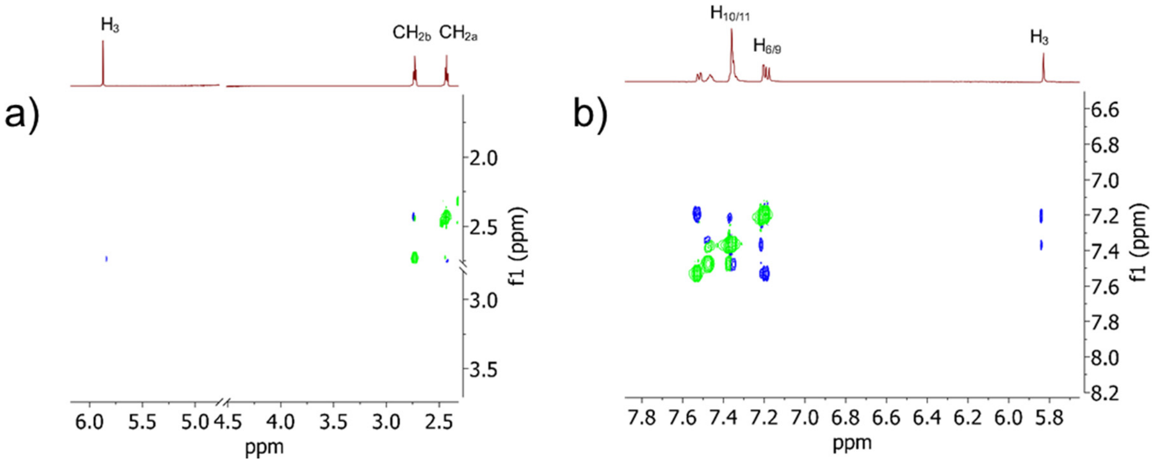

Figure S1. 2D ROESY map (600 MHz, K_2HPO_4/D_2O 50 mM, 25 °C, mix=0.3 s) expansion of OXEMIS (12 mM) for: low-frequency (a) and high-frequency signals (b).

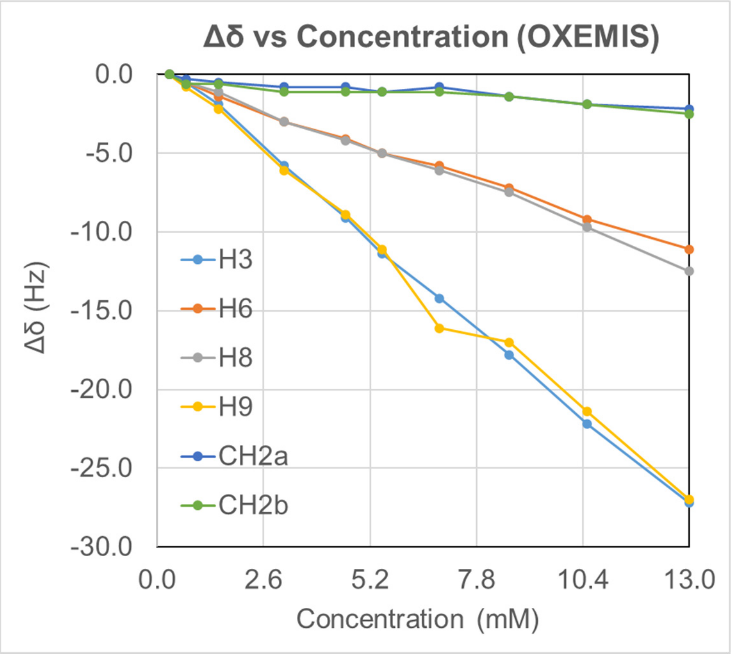

Figure S2. Dependence of OXEMIS ^{1}H NMR (600 MHz, K_2HPO_4/D_2O 50 mM, 25 °C) chemical shifts variations ($\Delta\delta=\delta_{[i\ mM]} - \delta_{[0.3\ mM]}$ Hz, with *i* from 0.3 to 13.7 mM) on concentration.

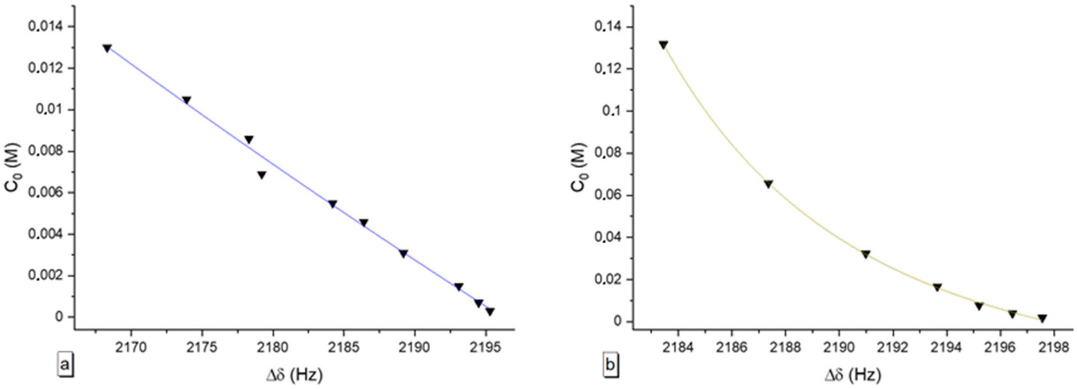

Figure S3. Curve describing the fitting of dilution data obtained for H_9 of OXEMIS in: a) D_2O (K_2HPO_4 50 mM) and b) $CDCl_3$.

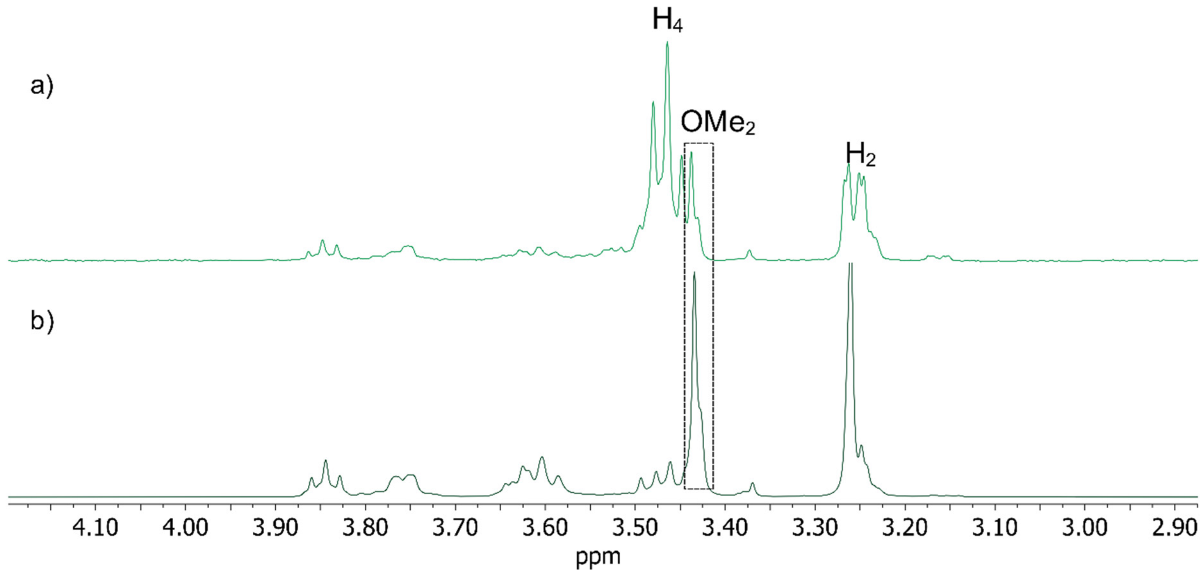

Figure S4. a) 1D ROESY spectrum of DIMEB (selected frequency H_1, mix=0.3 s) and b) ^1H NMR (600 MHz, K_2HPO_4/D_2O 50 mM, 25 °C) spectrum of DIMEB (12 mM).

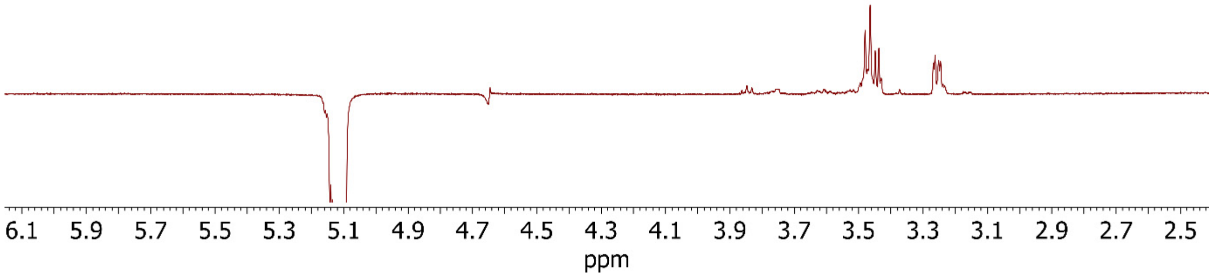

Figure S5. 1D ROESY (600 MHz, K_2HPO_4/D_2O 50 mM, 25 °C, mix=0.3 s) spectrum of DIMEB (12 mM), selected frequency H_1.

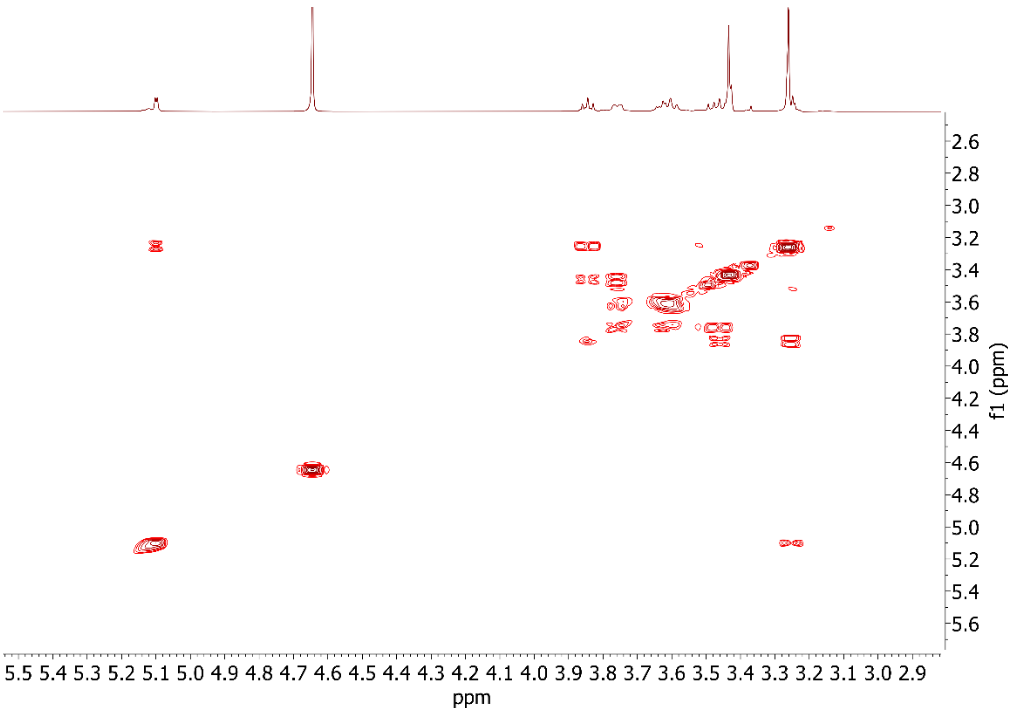

Figure S6. 2D COSY map (600 MHz, K_2HPO_4/D_2O 50 mM, 25 °C) of DIMEB (12 mM).

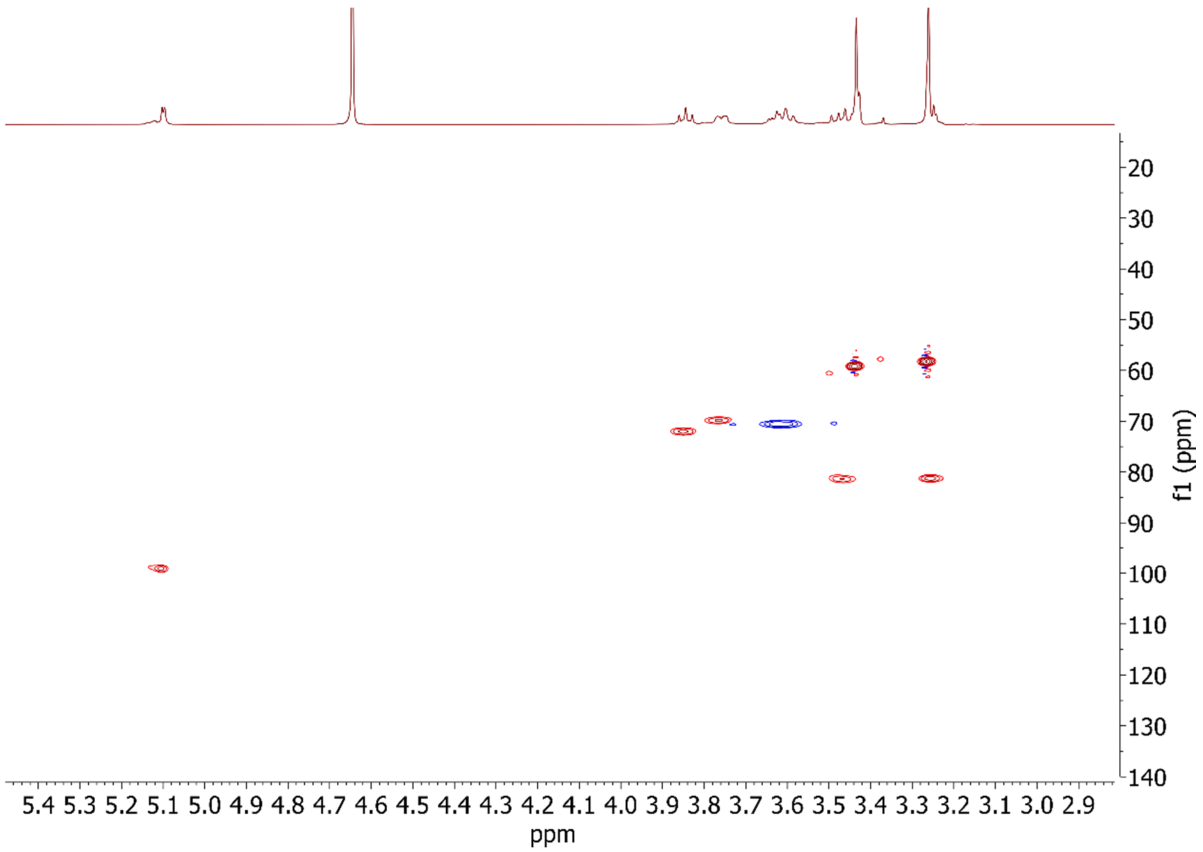

Figure S7. 2D HSQC map (600 MHz, K_2HPO_4/D_2O 50 mM, 25 °C) of DIMEB (12 mM).

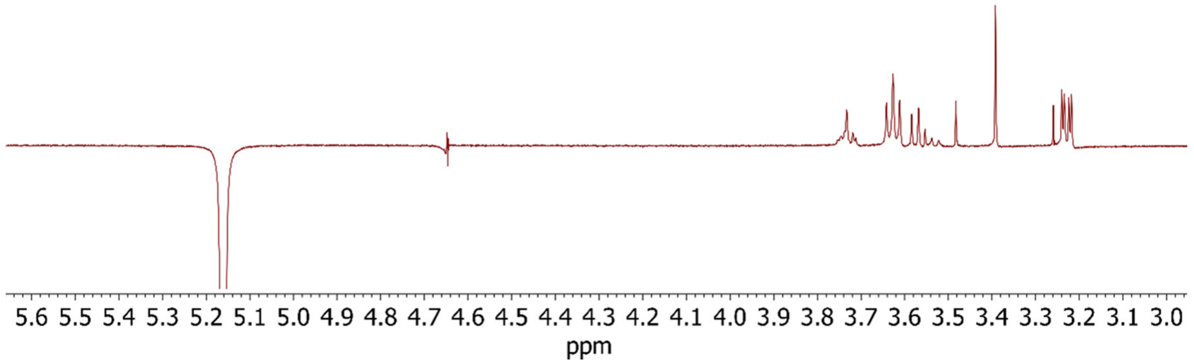

Figure S8. 1D ROESY (600 MHz, K_2HPO_4/D_2O 50 mM, 25 °C, mix=0.3 s) spectrum of TRIMEB (12 mM), selected frequency H_1.

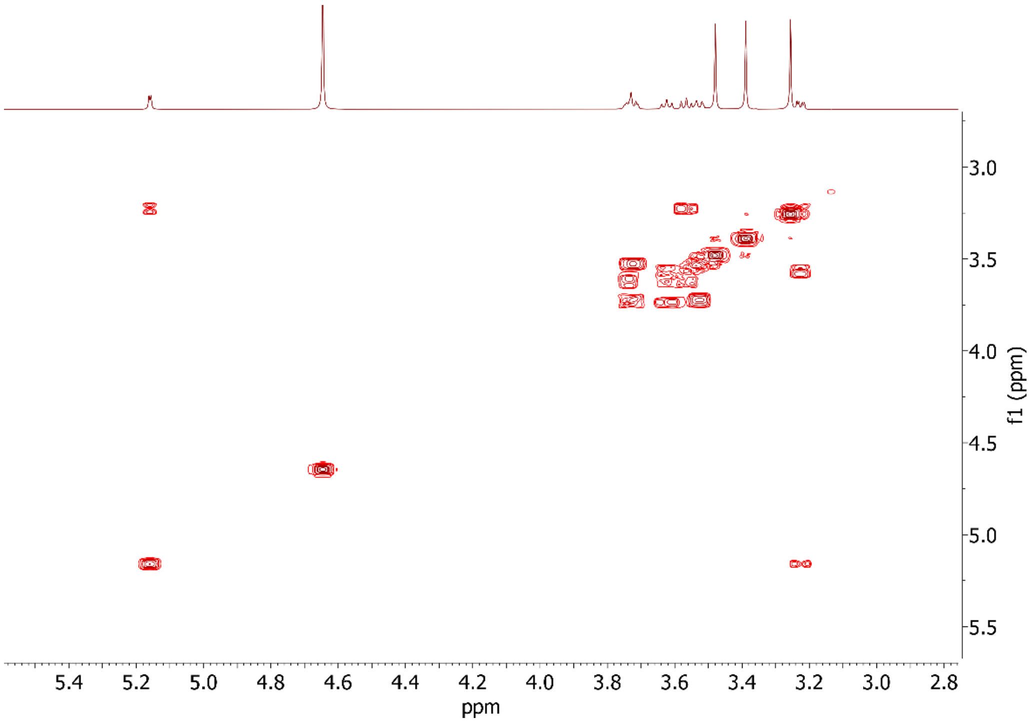

Figure S9. 2D COSY map (600 MHz, K_2HPO_4/D_2O 50 mM, 25 °C) of TRIMEB (12 mM).

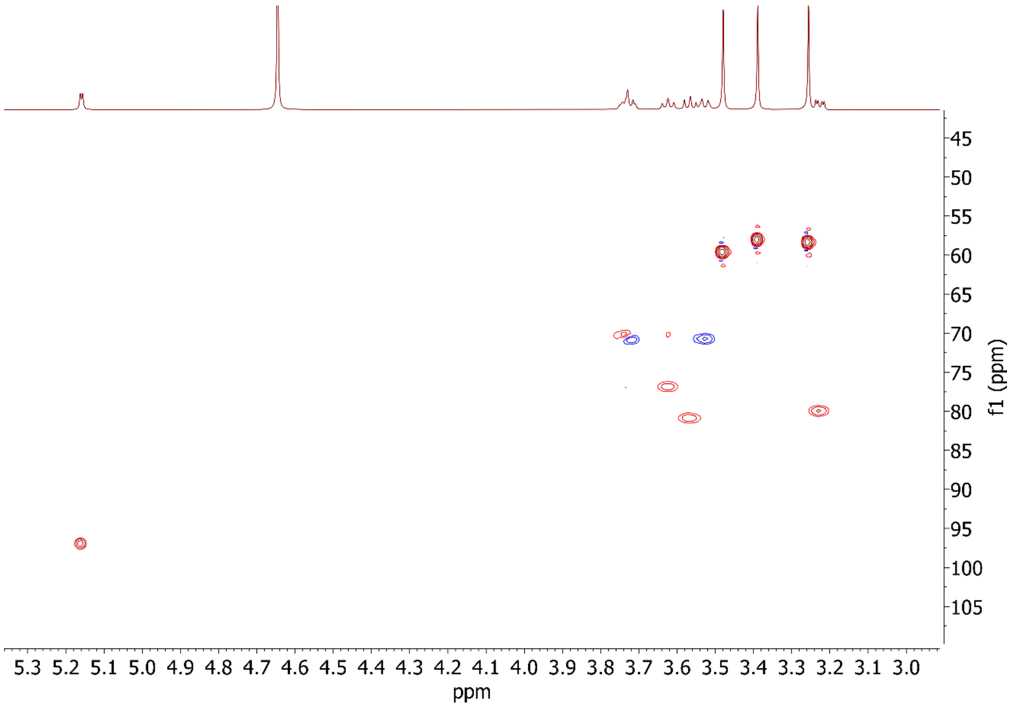

Figure S10. 2D HSQC map (600 MHz, K_2HPO_4/D_2O 50 mM, 25 °C) of TRIMEB (12 mM).

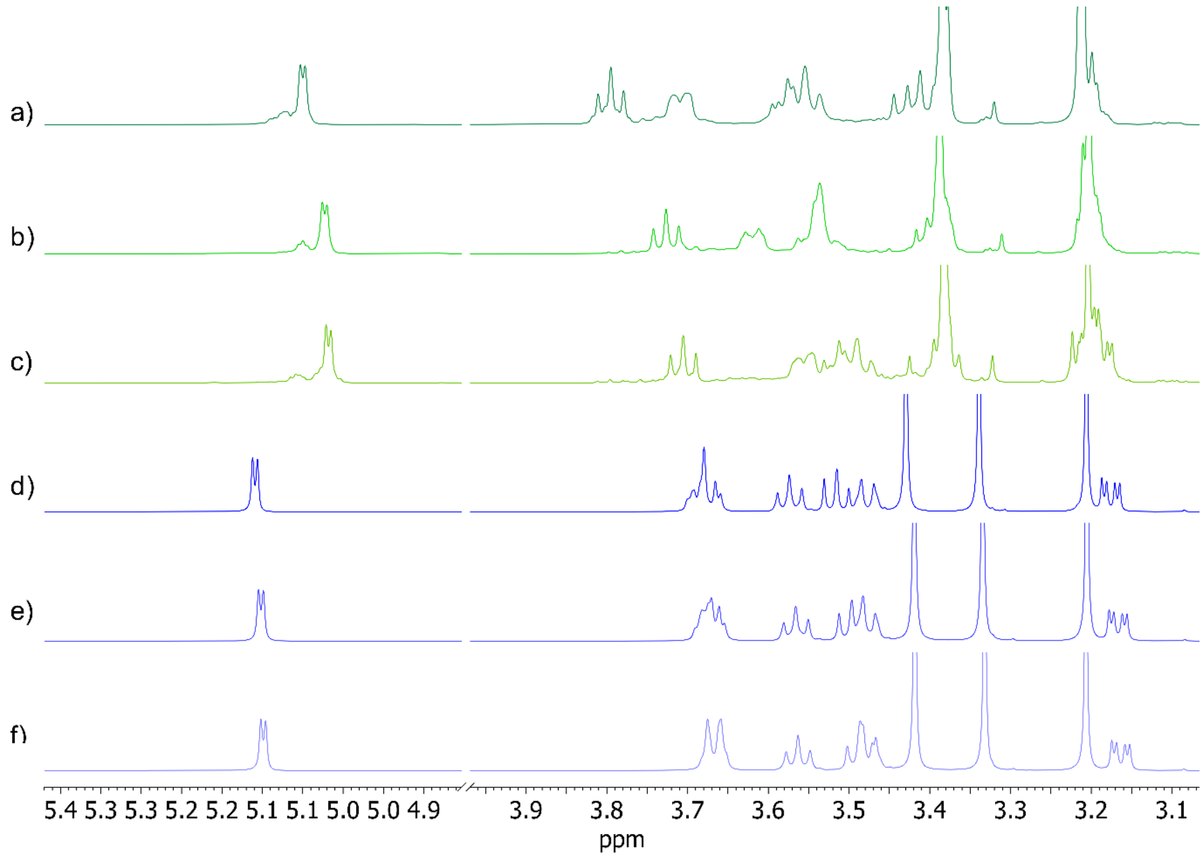

Figure S11. ^{1}H NMR (600 MHz, K_2HPO_4/D_2O 50 mM, 25 °C) spectra of cyclodextrins (12 mM): a) pure DIMEB, b) (*R*)-OXEMIS/DIMEB (1:2), c) (*S*)-OXEMIS/DIMEB (1:2), d) pure TRIMEB, e) (*R*)-OXEMIS/TRIMEB (1:2), and f) (*S*)-OXEMIS/TRIMEB (1:2).

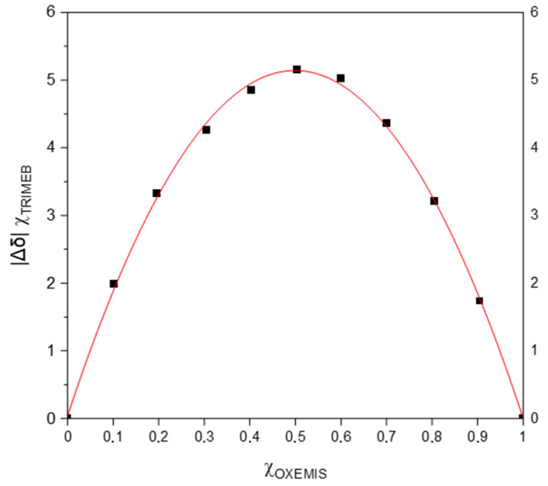

Figure S12. Job plot obtained for H_9 of (*S*)-OXEMIS in the presence of TRIMEB.

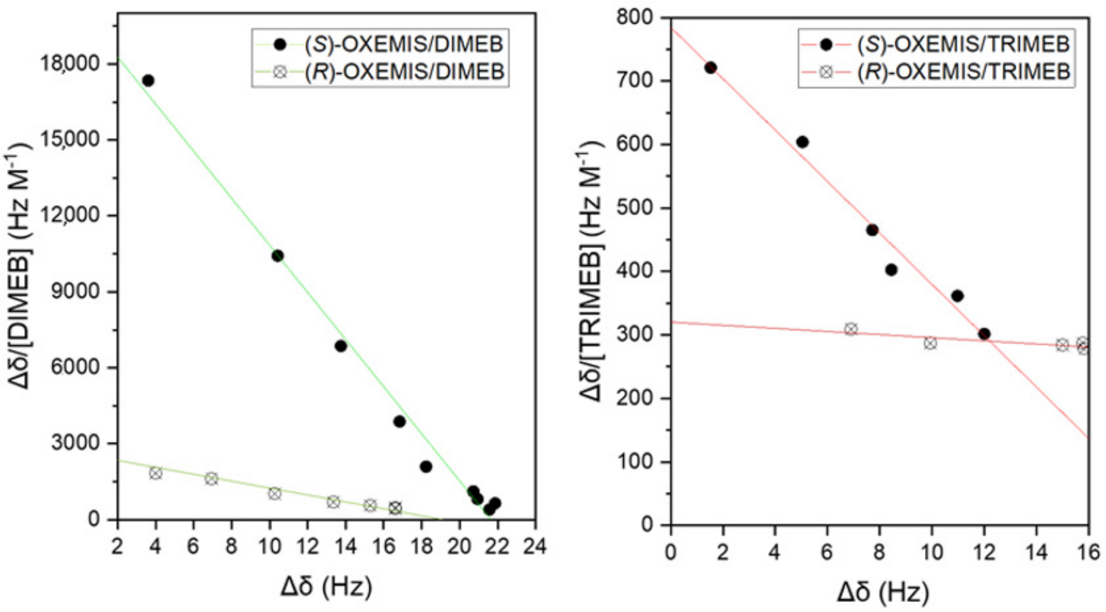

Figure S13. Linear fitting of titration data obtained for H_8 of (*R*)- and (*S*)-OXEMIS (D_2O, K_2HPO_4 50 mM) with DIMEB and TRIMEB: determination of association constant (K_a) by the Foster-Fyfe graphical method.
